# Forecasting one-day-forward wellness conditions for community-dwelling elderly with single lead short electrocardiogram signals

**DOI:** 10.1186/s12911-019-1012-8

**Published:** 2019-12-30

**Authors:** Xiaomao Fan, Yang Zhao, Hailiang Wang, Kwok Leung Tsui

**Affiliations:** 1 School of Data Science, City University of Hong Kong, Tat Chee Avenue, Kowloon, Hong Kong SAR, China; 2 Center for System Informatics, City University of Hong Kong, Tat Chee Avenue, Kowloon, Hong Kong SAR, China

**Keywords:** Elderly care, Wellness forecasting, Data mining, Deep learning

## Abstract

**Background:**

The accelerated growth of elderly population is creating a heavy burden to the healthcare system in many developed countries and regions. Electrocardiogram (ECG) analysis has been recognized as effective approach to cardiovascular disease diagnosis and widely utilized for monitoring personalized health conditions.

**Method:**

In this study, we present a novel approach to forecasting one-day-forward wellness conditions for community-dwelling elderly by analyzing single lead short ECG signals acquired from a station-based monitoring device. More specifically, exponentially weighted moving-average (EWMA) method is employed to eliminate the high-frequency noise from original signals at first. Then, Fisher-Yates normalization approach is used to adjust the self-evaluated wellness score distribution since the scores among different individuals are skewed. Finally, both deep learning-based and traditional machine learning-based methods are utilized for building wellness forecasting models.

**Results:**

The experiment results show that the deep learning-based methods achieve the best fitted forecasting performance, where the forecasting accuracy and *F* value are 93.21% and 91.98% respectively. The deep learning-based methods, with the merit of non-hand-crafted engineering, have superior wellness forecasting performance towards the competitive traditional machine learning-based methods.

**Conclusion:**

The developed approach in this paper is effective in wellness forecasting for community-dwelling elderly, which can provide insights in terms of implementing a cost-effective approach to informing healthcare provider about health conditions of elderly in advance and taking timely interventions to reduce the risk of malignant events.

## Background

The social and economic implications of aging population are becoming increasingly apparent in many countries and regions over the worldwide [1, 2]. Take Hong Kong for instance, the proportion of elderly aged 65 and over is projected to rise from 15% in 2014 to 36% in 2064 [3]. Since healthcare expenses increase significantly on average at the end of elderly people’s life, it is a heavy burden for local government and families to undertake the expenditures of medical services [4]. Fortunately, healthcare platforms can mitigate this kind of problems to a large degree, which provide daily healthcare monitoring services for elderly people via wearable and portable medical devices [5–7]. Most of them are centering on real-time monitoring rather than long-term forecasting for wellness conditions. However, long-term forecasting for wellness conditions has great potential in term of informing the associated healthcare provider about health conditions of elderly in advance and taking necessary interventions to reduce the possibility of malignant events. Therefore, developing an effective long-term forecasting method for wellness conditions of elderly has great significance in improving elderly care services.

In the past decades, many healthcare platforms for wellness monitoring have been developed, mainly including chronic diseases monitoring [5, 6, 8–10], cardiovascular diseases [11, 12], and general wellness monitoring [13–16]. He et al. [5, 6] proposed a six-layer healthcare cloud platform which collected physiological signals and vital signs from elderly and gave out a health evaluation report about hypertension, diabetes, and arrhythmias. Kara et al. [8] proposed a remote real-time health monitoring system. This system could provide heart conditions monitoring service and mitigate the problem of low doctor-to-patient ratio. Paradiso et al. [11] proposed a health monitoring system called WEALTHY which monitors individuals affected by cardiovascular diseases. Kailas et al. [13] proposed a general wellness system which could enable health-care professionals to master the wellness conditions by comprehensive real-time patient data. These healthcare platforms aforementioned process physiological data and vital signs on-line or off-line in the back-end, and deliver the corresponding healthcare reports of wellness conditions to the medical provider and cared individuals in real time or at fixed time. Thanks to the development of information technologies, these platforms become more and more stable and could provide more healthcare monitoring services. However, current healthcare platforms still have great deficiencies in forecasting long-term wellness conditions of elderly individuals. Therefore, researchers shifted their focus from healthcare monitoring to wellness conditions forecasting. Yu et al. [3] proposed a personalized healthcare monitoring platform to forecast one-day-forward wellness conditions for elderly. Integrating wearable data and vital signs from an all-in-one station-based monitoring device, they took advantage of machine learning tools to predict personal wellness conditions for elderly. However, their forecasting model is a highly personal data-dependent which could not provide an instant wellness forecasting service for other individuals.

Electrocardiogram (ECG) with the non-invasive and cost-effective merits is widely utilized to monitor heart health conditions such as atrial fibrillation [17], myocardial ischemia [18], and hypokalemia [19]. Due to the advanced technology of internet of things (IOT), single-lead ECG signals can be acquired conveniently by wearable/portable monitoring devices without the limits of time and locations [20]. In this study, we propose a one-day-forward forecasting method of wellness condition for community-dwelling elderly based on single lead short ECG signals. The proposed method mainly consists of exponentially weighted moving-average (EWMA) [21, 22] as a filter to remove high-frequency noises, Fisher-Yates normalization [3, 23] to mitigate the skewness of self-evaluated wellness scores, model selection based on deep learning and machine learning methods. Finally, the best fitted model validated by the visualization of learned features can be deployed into a healthcare platform to provide a forecasting wellness condition service.

The main contributions of this study are summarized as follows:

- We propose a novel framework using single lead ECG signals for forecasting one-day-forward wellness conditions of community-dwelling elderly using short ECG signals.
- Fisher-Yates normalization is utilized to adjust the self-evaluated wellness score distribution among different individuals.
- Based on deep learning and traditional machine learning methods, extensive wellness forecasting models are built and the best fitted forecasting model is selected for feature analysis and discussion of performance enhancement through the EWMA.
- The proposed framework can provide insights in terms of implementing a cost-effective approach to informing health conditions of elderly in advance and taking timely interventions to reduce the risk of malignant events.

The rest of this paper is organized as follows. The related work of forecasting methods is summarized in “Related work” section. In “Methods” section, both deep learning-based and traditional machine learning-based methods for forecasting elderly wellness conditions are described in detail. In “Results” section, experimental results are presented and the best forecasting model based on performance is selected. Feature visualization and optimization schemes are discussed in “Discussion” section. Finally, the conclusion is drawn in the last section.

## Related work

In this section, we review forecasting methods for temporal data particularly with applications to healthcare domain. These forecasting methods can be divided into two main categories: (i) traditional machine learning-based methods and (ii) deep learning-based methods.

For traditional machine learning-based forecasting methods, two representative approaches are support vector machine (SVM) and artificial neural network (ANN). Wu et al. [24] employed SVM to predict heart failure more than six months via vast electronic health records (EHR). The highest value of area under curve (AUC) for SVM is around 0.75. Santillana et al. [25] utilized the SVM to forecast estimates of influenza activity in America. Yu et al. [3] used the SVM to predict one-day-forward wellness conditions for elderly and achieved the forecasting accuracy of around 60%. Meanwhile, the ANN also obtained widely application in health care domain. Suryadevara et al. [26] took advantage of the ANN to forecast the behavior and wellness of elderly and deployed it into a healthcare prototype system. Srinivas et al. [27] employed the ANN to predict heart diseases like chest pain, stroke and heart attack. The prediction performances of these traditional machine learning-based methods are difficult to meet the precisely forecasting demands of elderly. So, researchers shifted their attention to cutting-edge deep learning-based forecasting methods.

In recent years, deep learning-based methods like recurrent neural network (RNN) has been achieved a big success in natural language processing, speech recognition, and machine translation [28–31]. Researchers also attempted to solve the problems in healthcare domain using these cutting-edge approaches [32–34]. Ma et al. [32] proposed an end-to-end simple recurrent neural network to model the temporality and high dimensionality of sequential EHR data to predict patients’ future health information. The experimental results based on two real world EHR datasets showed that their model improved the prediction accuracy significantly. Choi et al. [33] explored recurrent neural network whether improving initial diagnosis of heart failure compared to traditional machine learning-based approaches. Experimental results proved that recurrent neural network could leverage the temporal relations and improved the prediction performance of incident heart failure. Choi et al. [34] also proposed an interpretable forecasting model based on recurrent neural network. This deep model was tested on a large EHR dataset and demonstrated its superior prediction performance. Therefore, two popular deep learning-based approaches called long short-term memory network (LSTM) [35, 36] and bidirectional long short-term memory network (BiLSTM) [37] are utilized to forecast one-day-forward wellness conditions for elderly in this study. Meanwhile, two traditional machine learning-based methods of SVM and ANN are also employed for model selection.

## Methods

**Fig. 1:**
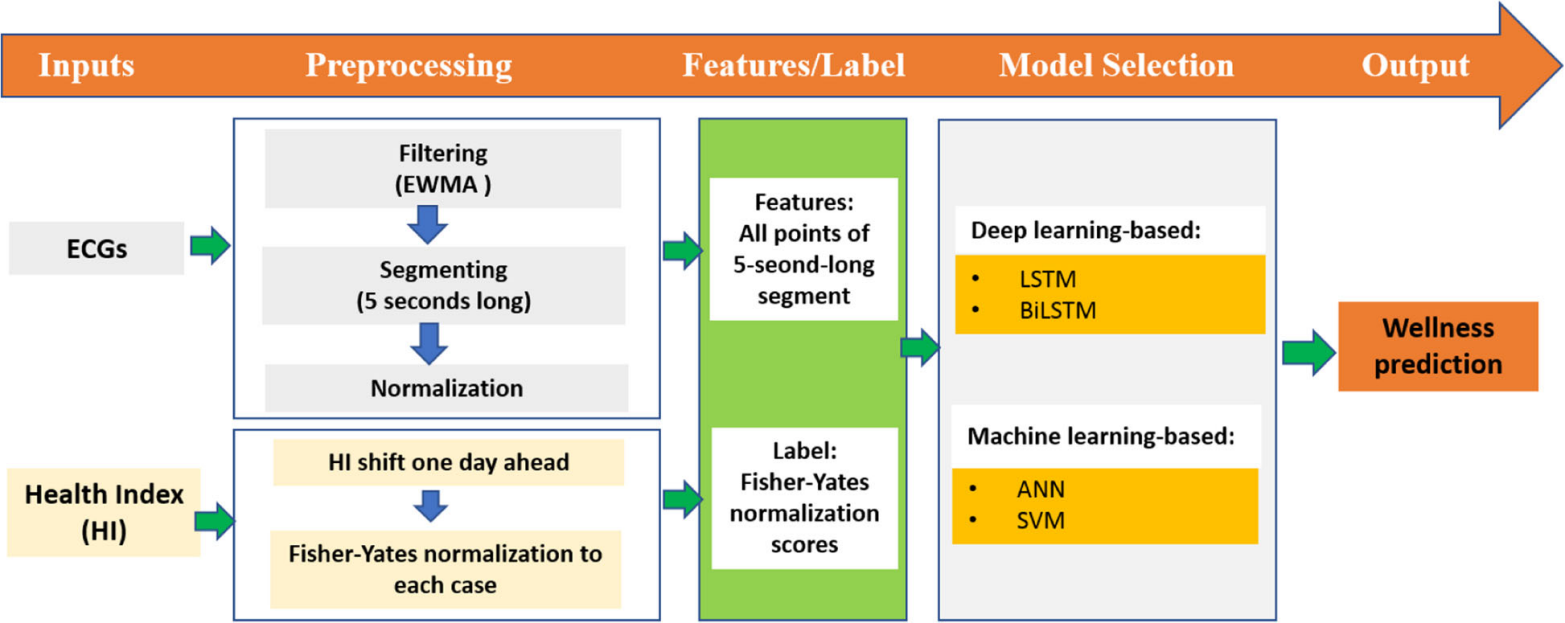
Overall procedure of the developed wellness prediction model. It mainly consists of data preprocessing and forecasting models. EWMA means exponentially weighted moving-average. LSTM means long short-term memory network. BiLSTM means bidirectional LSTM. ANN means artificial neural network. SVM means support vector machine

Figure 1 shows the whole pipeline of the proposed framework for forecasting one-day-forward wellness conditions of elderly. The proposed framework mainly consists of data preprocessing stage and model selection stage. More specifically, to eliminate the influence of high-frequency noise and skewness distribution, EWMA [21, 22] and Fisher-Yates normalization methods [3, 23] are employed in the procedure of data preprocessing. Meanwhile, to obtain a superior forecasting performance of one-day-forward wellness conditions, the state-of-the-art methods including deep learning-based and traditional machine learning-based methods are investigated for model selection. The details of these approaches are elaborated as follows.

### Problem Formulation

The task of this study is based on single lead short ECG signals to build a prediction model to forecast one-day-forward wellness conditions for elderly population. The input to the prediction model is an ECG signal $x_{i} = \left [x^{1}_{i}, x^{2}_{i}, \cdots, x^{n}_{i}\right ]$ with the length of *n*, where $x^{j}_{i}$ is the *j*-th element in the *i*-th ECG signal. Health index (HI) as the output consists of five health status categories from poor to excellent, and the corresponding score of HI is from 1 to 5. The HI scores as the ground truth are not suitable as the outputs of the prediction model directly since the HI scores are self-evaluated and subjective, which lead to serious skewness distribution. Therefore, the HI scores are preprocessed by Fisher-Yates normalization technique[3] which will be introduced in detail in the subsequent section. After Fisher-Yates normalization, the normalised HI scores *y* are dichotomized into better wellness condition (*y*=0) and worse wellness condition (*y*=1) based on a threshold value 0 (an example refers to Table 1). Therefore, the problem of this study is transformed into a classification problem. For a single instance in the training course, given training set *X*={*x*_1_,*x*_2_,⋯,*x*_*m*_} and ground truth wellnes condition set *Y*={*y*_1_,*y*_2_,⋯,*y*_*m*_}, the forecasting model aims to minimize the cross-entropy objective function as follows:

1 $$ L(X) = \frac{1}{m}\sum_{i=1}^{m}\left[y_{i}log\hat{y_{i}} + (1 - y_{i})log(1 - \hat{y_{i}})\right]   $$

**Table 1:** Example of health index vs. Fisher-Yates normalization score

Health index (HI)	3	4	5
Fisher-Yates normalization score	-1.332	-0.627	0.449
Binary wellness conditions	Worse	Worse	Better

where *y*_*i*_ is the ground-truth label, $\hat {y_{i}}$ is the predicted label, and *m* is the size of training set.

### Data preprocessing

#### Filtering

**Fig. 2:**
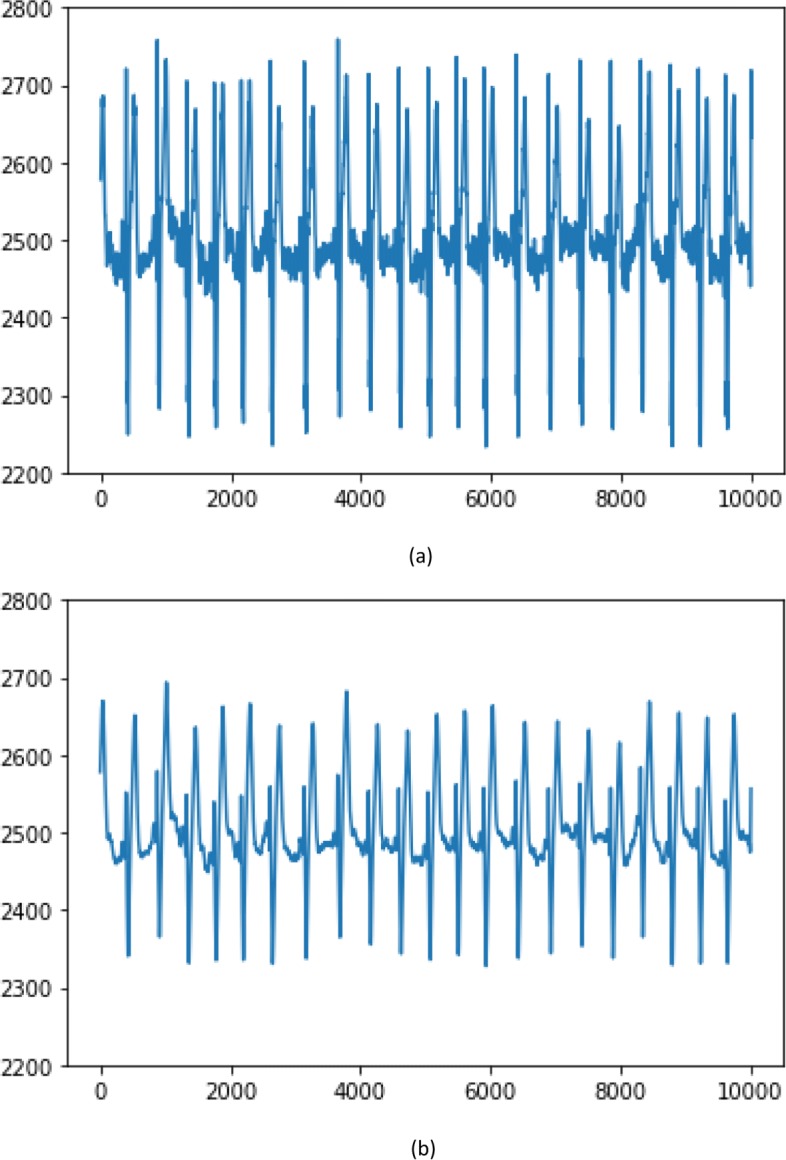
Filtering effect comparison of an original/EWMA filtered ECG signal. **a** Original ECG signal, **b** ECG signal through EWMA filtering

ECG signals acquired by a portable monitoring device are often contaminated by variety of noises. The EWMA [38] as a low-pass filter is utilized to cancel the high-frequency noise. The EWMA is a moving average with exponential diminishing with time, which is somewhat related to the number of points in a moving average. The EWMA can be defined as:

2 $$ EWMA_{t} = \sum_{i=1}^{n}(1 - \alpha)^{n - i}x_{i}   $$

where *EWMA*_*t*_ is the output of the *t*-th time point with the window size *n* of a moving average. $\alpha = \frac {2}{1 + n}$, which indicates the rate of weight decline. *n* refers to the number of points in a moving average. *x*_*i*_ is the *i*-th point in the window. As shown in Eq. 2, one can observe that recent points in a moving average have higher weighting, far previous points have almost no weight. As the number of points in a moving average increases, the EWMA filter can produce a smoother signal with larger response lag. In this study, *n* is set to a popular value 40 in time series domain. An ECG signal was sampled out from training set to cancel high-frequency noise with the EWMA. As shown in Fig. 2, the ECG signal through EWMA filtering has reduced random noise greatly, which is helpful for improving forecasting performance of subsequent classifiers.

#### Segmentation

The length of input ECG signals acquired from elderly nursing center varies from 20 seconds to 25 seconds. However, most of deep learning-based and traditional machine learning-based methods require fixed input length. In this study, an ECG signal is segmented into segments of 5 seconds long with stride 1 second [17], which includes about 4 to 9 heartbeats. Take a 20-second-long ECG signal for example, this signal can be segmented into 15 5-second-long ECG signals with aforementioned scheme. It can greatly increase the size of training set, which would enhance the forecasting performance of deep learning-based methods.

#### Normalization

The amplitude of ECG signals vary largely from different individuals, even for the same individual with different time. In practice, normalization for input data help machine learning methods to converge quickly, particularly for deep learning-based methods [17]. Regarding the ground truth self-evaluated HI scores, different elderly may provide different HI score even if they have the similar feeling of wellness condition. Using a normalization scheme are necessary to balance the bias of subjective feeling. In this study, min-max normalization technique is used for normalizing input ECG signals as well as Fisher-Yates normalization technique [3] for ground truth label HI.

**Fig. 3:**
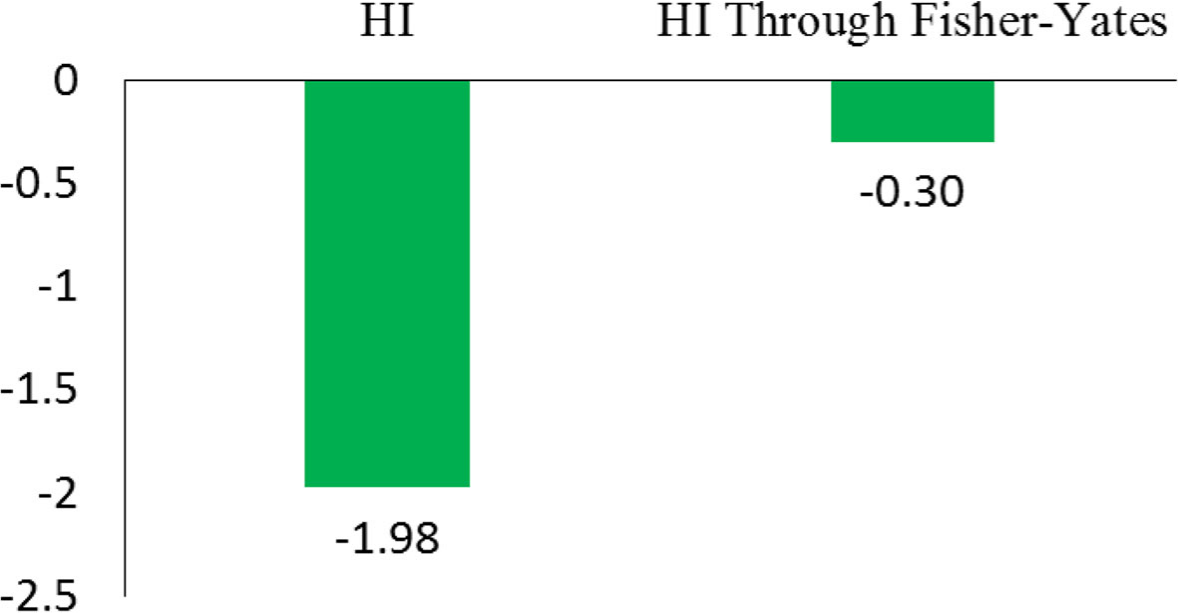
Skewness comparison between original HI and HI through Fisher-Yates normalization

- Min-max normalization: this technology is one kind of common used statistic normalizing tools, which maps all of the values into range [0,1]. The min-max normalization can be defined as:
  3 $$ X_{new} = \frac{X - X_{min}}{X_{max} - X_{min}}   $$
  where *X*_*new*_ means the output of min-max normalization. *X*_*min*_ means the minimum value of the input ECG signal. *X*_*max*_ means the maximum value of the ECG signal.
- Fisher-Yates normalization: generally, Fisher-Yates normalization has intrinsic ability to remove the skewness of original data, which is pretty appropriate for HI transformation. Suppose *x*_*ij*_ is HI score of the *i*-th day of the *j*-th elderly. Let *r*_*ij*_ be the rank of the *i*-th score among the assessment course of the *j*-th elderly, 1≦*i*≦*I* and 1≦*j*≦*P*. Then *x*_*ij*_ can be replaced by Ψ^−1^(.), which is defined as:
  4 $$ FY_{norm} (x_{ij}) = \varPsi^{-1}\left(\frac{r_{ij}}{I + 1}\right)   $$
  where *FY*_*norm*_ is an array of Fisher-Yates normalization scores. To simplify the problem of forecasting one-day-forward wellness condition, the *FY*_*norm*_ scores of HI are mapped into binary values 0 and 1. More specifically, *FY*_*norm*_ scores greater than 0 are mapped as value 0 representing better wellness condition, otherwise as value 1 representing worse wellness condition. As shown in Table 1, it is an instance of health index vs. Fisher-Yates normalization score from an elderly. Since the recruited elderly only gave three of self-evaluated health index, this example presents the corresponding Fisher-Yates normalization scores and binary wellness conditions. One can observe that HI score 3 and 4 are mapped as worse condition as well as HI score 5 are mapped as better condition after Fisher-Yates normalization. By means of this process, Fig. 3 shows that the skewness in HI scores is almost removed.

### Classification methods

In this study, the problem of forecasting one-day-forward wellness conditions can be transformed into a typical binary classification problem by shifting HI score one-day-ahead for each elderly. Since the input ECG signal is a sequence, we utilize both deep learning-based and traditional machine learning-based methods for forecasting one-day-forward wellness conditions by using short ECG signals. The deep learning-based methods used are LSTM and BiLSTM. Meanwhile, the employed traditional machine learning-based methods include ANN and SVM. These methods have been widely applied in healthcare domain in recent published literature, which are described in detail as follows:

#### Deep Learning-based methods

**Fig. 4:**
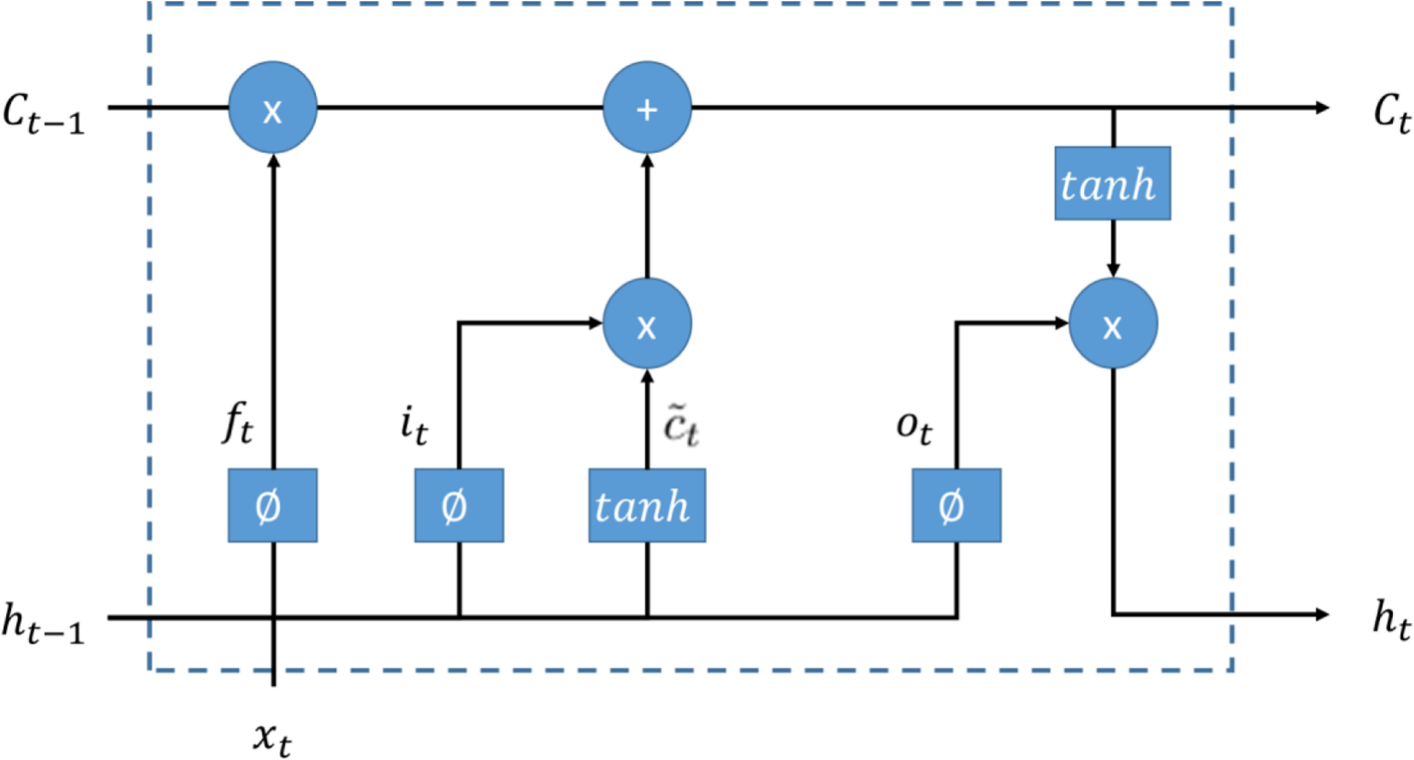
The LSTM unit. A LSTM unit consists forget gate, input gate, output gate, and input. The sigmoid function *ϕ* with range from 0 to 1 mimics the gate open and close

**Fig. 5:**
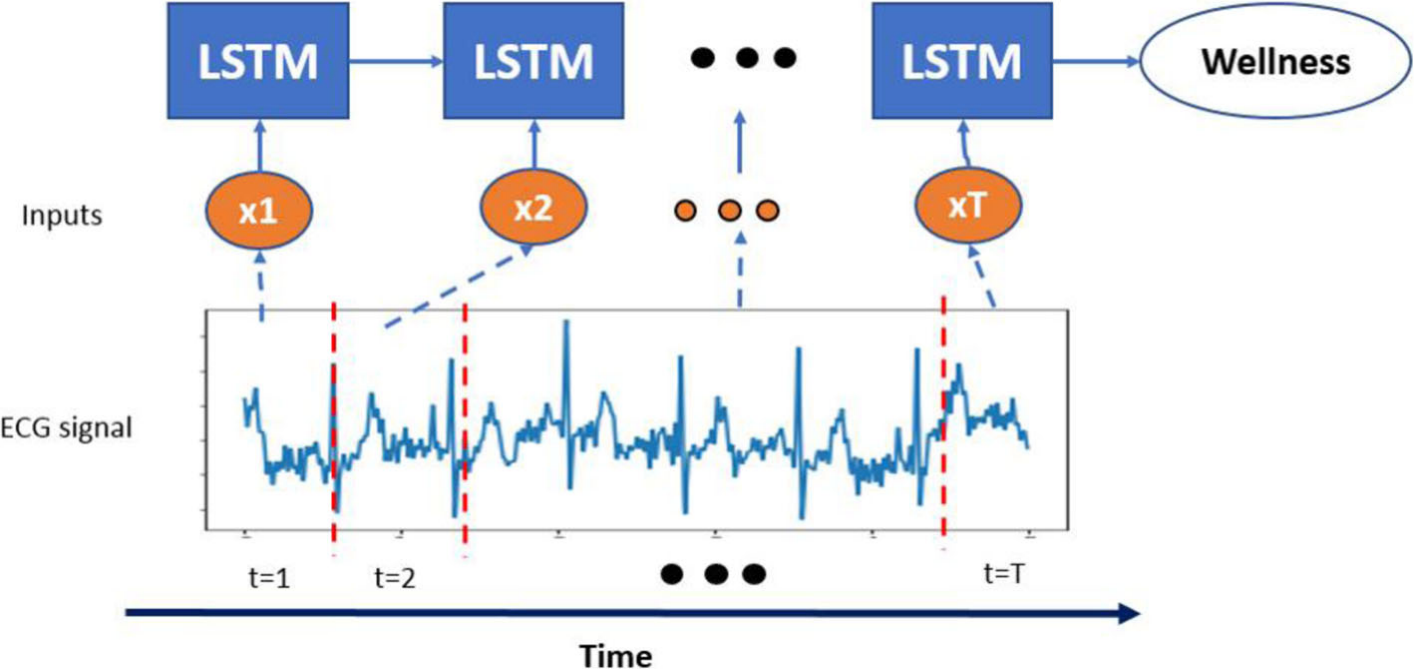
The LSTM network unrolled along the time axis. An ECG signal is segmented into *T* segments, each segment is fed into one LSTM unit
  6 $$\begin{array}{*{20}l} f_{t} &= \phi (W_{if} \cdot x_{t} + b_{if} + W_{hf} \cdot h_{t-1} + b_{hf}) \end{array} $$
  7 $$\begin{array}{*{20}l} o_{t} &= \phi (W_{io} \cdot x_{t} + b_{io} + W_{ho} \cdot h_{t-1} + b_{ho}) \end{array} $$
  8 $$\begin{array}{*{20}l} \tilde{c}_{t} &= \tanh(W_{i\tilde{c}} \cdot x_{t} + b_{i\tilde{c}} + W_{h\tilde{c}} \cdot h_{t-1} + b_{h\tilde{c}}) \end{array} $$
  9 $$\begin{array}{*{20}l} c_{t} &= f_{t} \cdot c_{t-1} + i_{t} \cdot \tilde{c}_{t} \end{array} $$
  10 $$\begin{array}{*{20}l} h_{t} &= o_{t} \cdot \tanh(c_{t}) \end{array} $$
  where *c*_*t*_ is the cell neuron at time *t*, *h*_*t*_ is the hidden neuron at time *t*, *h*_*t*−1_ is the hidden neuron at time *t*−1, and *i*_*t*_, *f*_*t*_, *o*_*t*_, $\tilde {c}_{t}$ are the input gate, forget gate, output gate, and cell neuron, respectively. *W* and *b* are the connected weights and bias among the input, cell neuron, and hidden neuron. *ϕ*(·) is a sigmoid function. As for the final output wellness condition *y*, it can be obtained via a Softmax function of the last output neuron *h*_*T*_ of the LSTM, which can be described as follows:
  11 $$ y = Softmax(h_{T})   $$
- BiLSTM: the BiLSTM is a variant from the LSTM which is widely used in processing sequence data. To capture the global pattern in a long-term sequence, the BiLSTM has two hidden layers to store history information from opposite directions to the same output. Figure 6 show the architecture of the BiLSTM network unrolled along the time axis. Like the LSTM, the BiLSTM also has three of input gate, forget gate, and output gate in each LSTM unit which are described in the previous section. In this study, we concatenate the last hidden neuron from both forward propagation and backward propagation layers as the concatenated hidden neuron *h*_*con*_. Subsequently, a Softmax layer is connected to forecast the wellness condition. These can be described from the following functions:
  12 $$\begin{array}{*{20}l} h_{con} &= Concatenate(h_{T-foreward}, h_{1-backward}) \end{array} $$

- LSTM: the LSTM is a special kind of recurrent neural network, which is proposed to solve the problem of gradient dispersion in the traditional recurrent neural network (RNN). The LSTM is different from the RNN mainly in that it adds memory cells (also named LSTM units) with three gates to the algorithm to judge whether the information is useful or not. These three gates are the input gate, the forget gate, and the output gate, which enable the LSTM units to read, write, reset, and update historical information over long distance. As shown in Fig. 4, when a piece of information enters the LSTM unit, the input gate determines how much information of the input is updated into the memory cell. And the forget gate controls how much information kept for memory cell. Only a part of historical memory information that is helpful for final task will be left, the rest parts of historical memory information are discarded through the forget gate. The output gate, the control mechanism like the input gate and forget gate, determines how much information in the memory cell outputs. These three control gates employ individual sigmoid function with a range between 0 and 1 to mimic the gate open and close, which means how much percentage of information is kept for next process. The gate techniques empower the LSTM the capability of learning hidden pattern from a long-term sequence. Figure 5 shows the architecture of the LSTM we employ in this study. More specifically, an ECG signal with fixed length is segmented into *T* sequences, each sequence *x*_*t*_ is fed into one LSTM unit. For a LSTM unit in each time step of the input ECG signal, it can be defined as the following functions:
  5 $$\begin{array}{*{20}l} i_{t} &= \phi (W_{ii} \cdot x_{t} + b_{ii} + W_{hi} \cdot h_{t-1} + b_{hi}) \end{array} $$

**Fig. 6:**
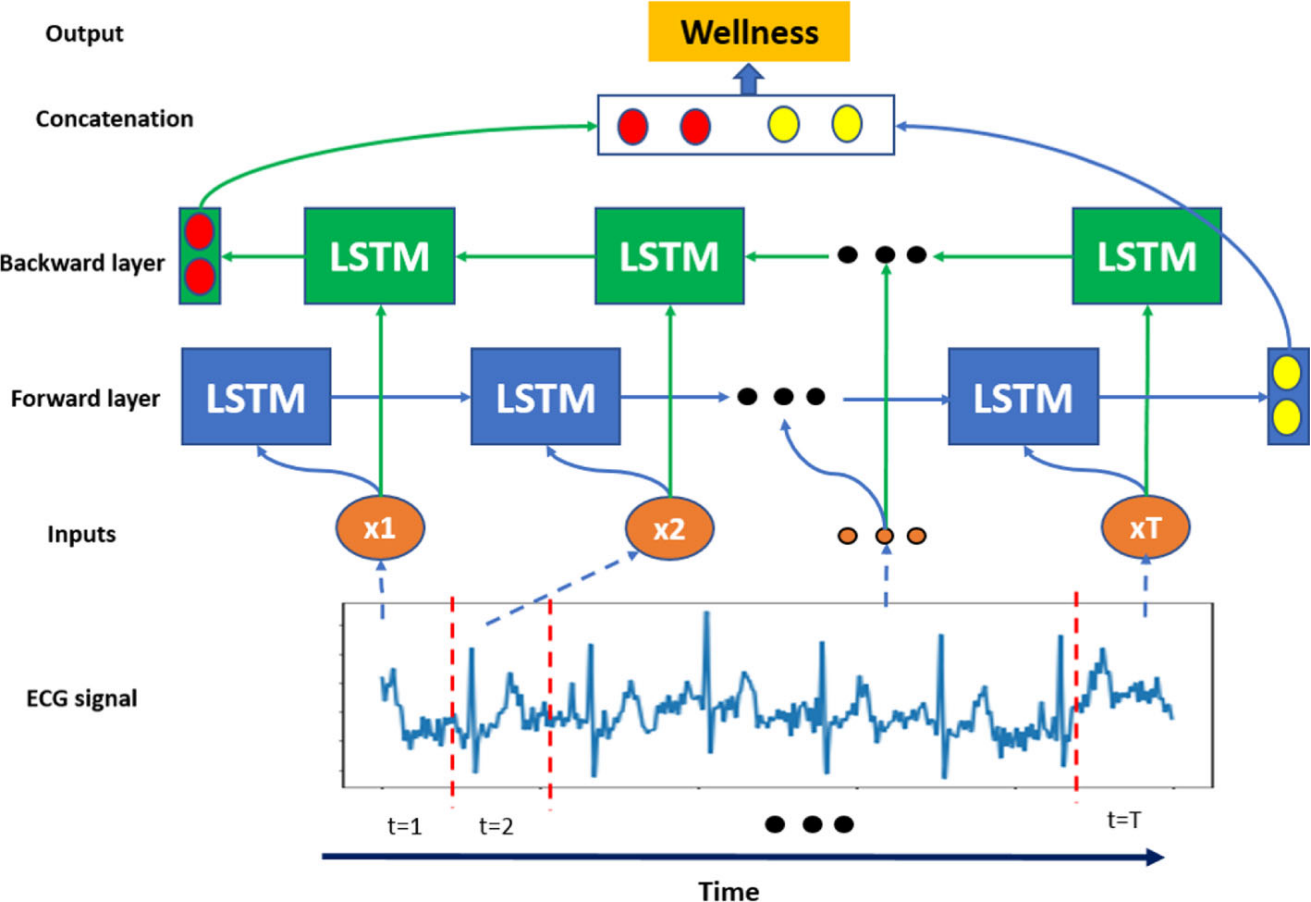
The BiLSTM network unrolled along the time axis. LSTM units in blue constitute the forward layer as well as LSTM units in green constitute the backward layer
  13 $$\begin{array}{*{20}l} y &= Softmax(h_{con}) \end{array} $$
  where *h*_*T*−*foreward*_ is the last hidden neuron in the forward propagation hidden layer, *h*_1−*backward*_ is the last hidden neuron in the backward propagation hidden layer, and *y* is one-day-forward wellness condition.

#### Traditional machine learning-based methods

**Fig. 7:**
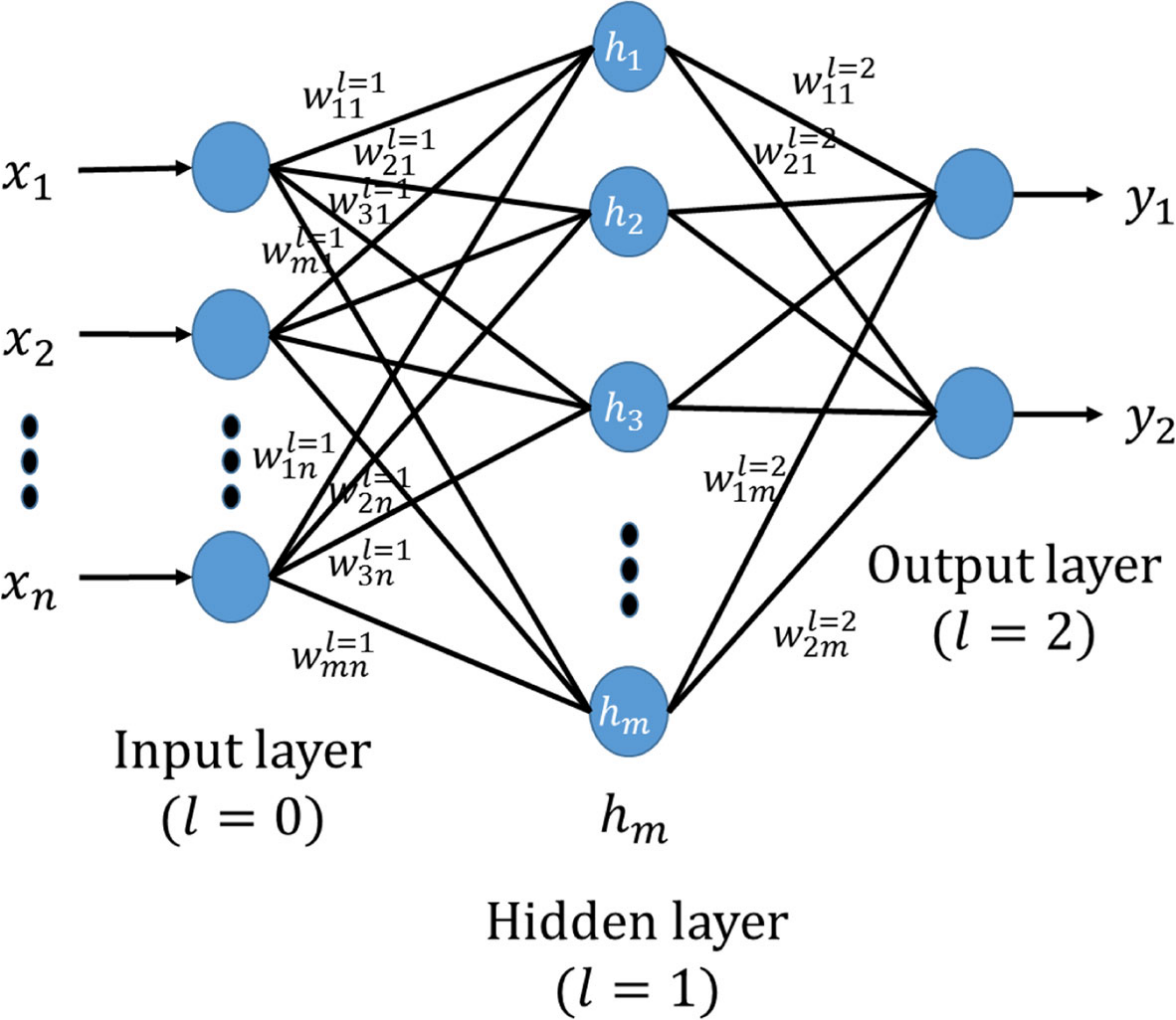
The architecture of the single-layer ANN. It is a simple architecture of the ANN, including an input layer, a hidden layer, and an output layer
  15 $$\begin{array}{*{20}l} y_{i} &= \sum_{j=1}^{m} w_{ji}^{l=2} \cdot h_{i} + b_{i}^{l=2} \end{array} $$
  where *h*_*i*_ is the *i*-th hidden unit, $w_{ji}^{l}$ is the *l*-th layer weight from the *i*-th unit in the *l*−1-th layer such as input unit to the *j*-th unit in the *l*-th unit such as hidden unit, $b_{i}^{l}$ is the *i*-th correponding bias in the *l*-th layer. Function *σ* is the activation function *sigmoid*. *y*_*i*_ is the *i*-th output unit of one-day-forward wellness condition in this study. *n* is the input size and *m* is the number of hidden units.
- SVM: the SVM is a generalized supervised linear classifier that carries out binary classification. Its decision boundary is the maximum-margin hyperplane that is solved for learning samples. Given training set *D*={(*x*_1_,*y*_1_),(*x*_2_,*y*_2_),(*x*_3_,*y*_3_),⋯,(*x*_*n*_,*y*_*n*_)}, *y*_*i*_∈{−1,1}, the hyperplane as shown in Fig. 8 can be described in equation as follows:
  16 $$ W^{T} \cdot x + b = 0   $$

- ANN: the ANN is a simulated biological neural networks formed by several very simple processing hidden units connected with each other in some way. The ANN model consists of a large volume of hidden units. Each unit represents a specific output function called activation function. Each connection between two hidden units represents a weighted value of the signal passing through the connection, called a weight *w*, which is equivalent to the memory of the artificial neural network. Figure 7 shows a simple architecture of the ANN with an input layer, a hidden layer, and an output layer. The ANN is suitable for regression and classification problem and can be described as follows:
  14 $$\begin{array}{*{20}l} h_{i} &= \sigma\left(\sum_{j=1}^{n} w_{ji}^{l=1} \cdot x_{i} + b_{i}^{l=1}\right) \end{array} $$

**Fig. 8:**
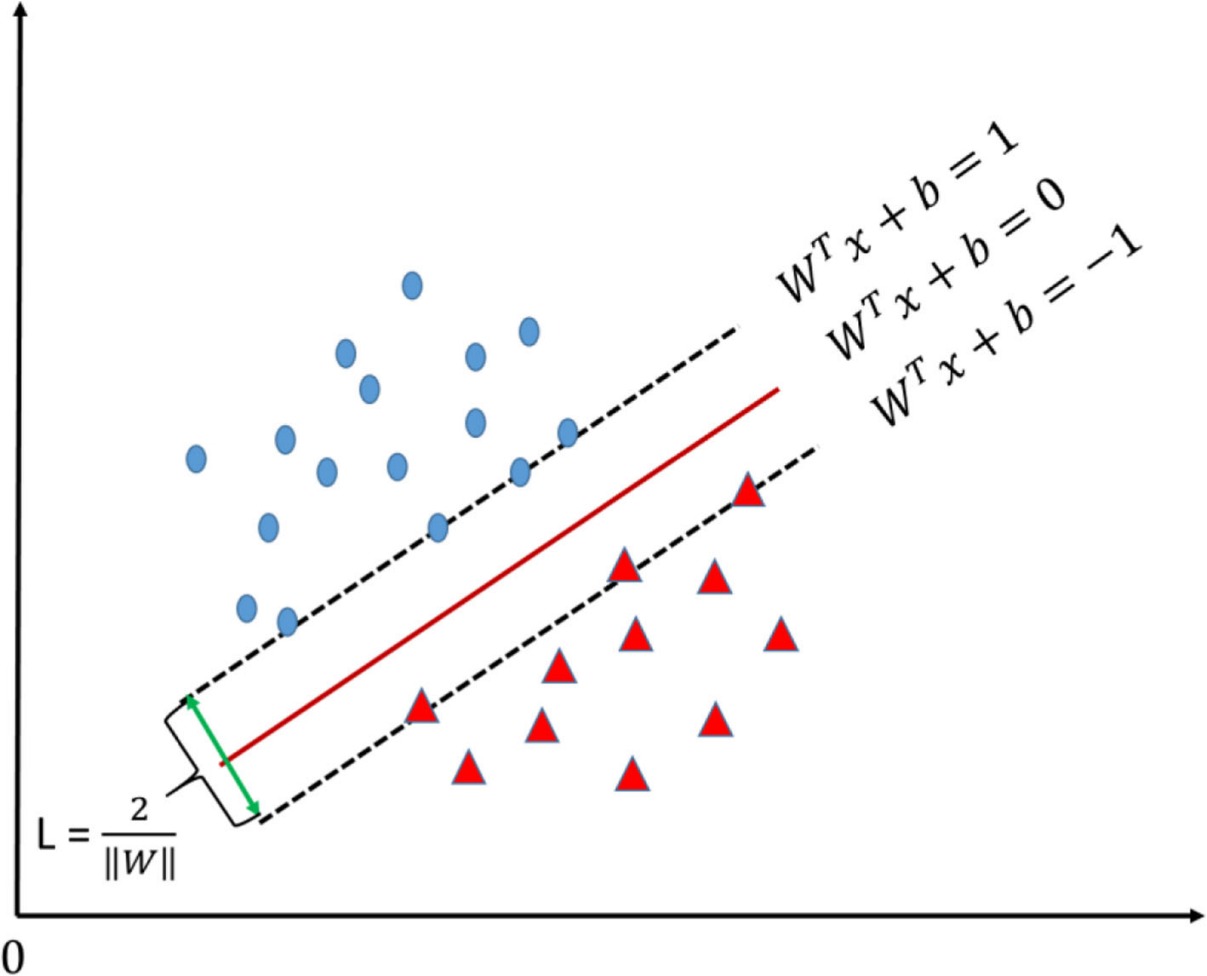
Support vectors. It is a example of SVM, which demonstrates support vectors and maximum margin
  where *x*_*i*_ refers to ECG segment, *y*_*i*_ refers to wellness condition which has two categories: *y*_*i*_=1 represents worse wellness condition and *y*_*i*_=−1 represents better wellness condition. *W* is the normal vector, which determines the direction of the hyperplane, and *b* is the displacement, which determines the distance between the hyperplane and the origin. The objective of SVM is to maximize the margin *L* between two support hyperplanes, which can be described as follows:
  17 $$\begin{array}{*{20}l} &Max \quad L = \frac{2}{\parallel W \parallel} \end{array} $$
  18 $$\begin{array}{*{20}l} & \quad s.t. \quad y_{i} \cdot (W^{T}x_{i} + b) \geq 1, i = 1, 2, 3, \cdots n \end{array} $$
  The SVM uses hinge loss function to calculate empirical risk and has added regularization term into the solving system to optimize structural risk, which is a classifier with sparsity and robustness. The SVM can conduct non-linear classification through kernel method, which is one of the common kernel learning methods. In this study, we use the widely used kernel function radial basis function (RBF) as the SVM kernel function, which can be described as:
  19 $$ k\left(x_{1}, x_{2}\right) = exp \left(-\frac{\parallel x_{1} - x_{2} \parallel^{2}}{2\sigma^{2}}\right)   $$
  where *X* is the input, and *σ* is the standard deviation to control the shape of mapping features.

### Classification performance metrics

All of the forecasting models are tested on the independent data set and evaluated by a set of classification performance metrics, which are critical important to assess the forecasting models’ performance [3]. In this study, the classification performance metrics mainly consist of Recall (*REC* for short), Precision (*PRE* for short), false prediction rate (*FPR* for short), and overall accuracy (*ACC* for short). A metric of a test’s accuracy called *Fscore* also be used as a trade-off evaluation score between recall *REC* and precision *PRE*. These performance metrics can be defined as:

20 $$\begin{array}{*{20}l} REC &= \frac{TP}{TP + FN} \end{array} $$

21 $$\begin{array}{*{20}l} PRE &= \frac{TP}{TP + FP} \end{array} $$

22 $$\begin{array}{*{20}l} FPR &= \frac{FP}{FP + TN} \end{array} $$

23 $$\begin{array}{*{20}l} ACC &= \frac{TP + TN}{TP + FP + TN + FN} \end{array} $$

24 $$\begin{array}{*{20}l} Fscore &= 2\cdot\frac{ REC \cdot PRE}{REC + PRE} \end{array} $$

where *TP* refers to the number of correctly predicted entries with worse wellness condition. *TN* refers to the number of correctly predicted entries with better wellness condition. *FP* refers to the number of wrong predicted entries with worse wellness condition. *FN* refers to the number of wrong predicted entries with better wellness condition. In addition to the aforementioned performance metrics, the value of area under receiver operating characteristic curve (AUC for short) is also employed to measure the advantage and disadvantage of forecasting models in this study. According to the definition, the AUC value can be obtained by summing the area of each part under the receiver operating characteristic curve.

## Results

### Experimental environment

All the experiments in this study run on a powerful computing server equipped with four 4-core Intel(R) Xeon W-2102 CPUs at 2.90GHz, 64 GB memories, and two 128-core NVIDIA GP104GL (Quadro P5000) GPUs at 1.73Hz. A prevailing linux system Ubuntu 16.04.6 LTS is installed in the computing server, with a deep learning framework Pytorch 1.0.1 for deep neural networks training and testing.

### Data source

In this study, eleven elderly persons (age: 76 ±7.8 and gender: 9 females and 2 males) were recruited from an elderly nursing home of Hong Kong for wellness condition evaluation. All participants gave their written informed consent. The data collection period lasted for three months. During this period, all the participants were invited to join the daily non-invasive assessments with a commercial healthcare monitoring device TeleMedCare [39]. The TeleMedCare is a station-based healthcare monitoring device, which can acquire elderly vital signs like systolic blood pressure, diastolic blood pressure, single lead ECG signals. The length of collected ECG signals with sampling frequency 500 Hz is from 20 to 25 seconds. Meanwhile, a 5-point self-evaluated scoring system was utilized to assess wellness conditions of the participants according to a tailor-made questionnaires [40]. As shown in Table 2, each subject was requested to self-evaluated wellness conditions and gave out the appropriate associated HI score immediately when the TeleMedCare completed their physiological data collection. In order to guarantee the data quality, the process of data collection were done under the guidance of trained and qualified research staffs at the elderly nursing center around 11 am during the assessment period. Due to personal affairs of elderly subjects like ill in hospital during the course of assessment, the associated vital signs and physiological data were missed. Excluding aforementioned missing observations, total 383 including ECG signals and HI scores can be used for wellness forecasting model.

**Table 2:** Self-evaluated health index for wellness conditions

Health index (HI)	Wellness condition description
1	Poor
2	Fair
3	Good
4	Very good
5	Excellent

### Classification performance

In this section, 10-fold cross validation is utilized to evaluate the forecasting models’ performance. We implement the forecasting models of one-day-forward wellness conditions based on both deep learning-based methods and traditional machine learning-based methods with grid search scheme to obtain the optimized parameters. For the ANN model, there are two superior parameters of hidden size *h* and learning rate *η* to optimize. In order to obtain the best forecasting performance of the ANN, we take advantage of grid search technique to choose the optimized hidden size, which ranges from 100 to 1000 with an increase step of 10. The initial learning rate *η*_0_ is set to 0.01, which decays automatically by a factor 0.1 every 100 epochs. The total iterated epochs *N* are set to 500 in this paper. As for the SVM model, we also tune two superior parameters of penalty parameter *C* and kernel coefficient *Γ* ranging from 10^−8^ to 10^8^ with a 10 times increase step. While the optimized superior parameters of LSTM and BiLSTM are the same, which consist of the learning rate *η*, hidden size *h*, and input size *h*. The learning rate *η*_0_ is set to 0.6 and decays automatically when the error loss *ε* has stopped improving every 10 epochs. The learning rate is reduced to *η* times a factor *f* which is set to 0.1. With respect to the optimized experimental model configuration, please refer to Table 3.

**Table 3:** Configuration of the forecasting models for wellness conditions

Models		Optimized superior parameters
Deep learning-based	LSTM	*h*: 256, *i*: 100, *η*_0_: 0.6, *f*: 0.1. Optimized scheme of *η*: *η*·*f* when *ε* not improving every 10 epochs.
	BiLSTM	*h*: 256, *i*: 100, *η*_0_: 0.6, *f*: 0.1. Optimized scheme of *η*: *η*·*f* when *ε* not improving every 10 epochs.
Machine learning-based	ANN	*h*: 100, *η*_0_: 0.01, *f*: 0.01. Optimized scheme of *η*: *η*·*f* per 100 epochs
	SVM	*C*: 10, *Γ*: 0.01, Kernel: RBF Tolerance for stopping criterion: 10^−3^

**Fig. 9:**
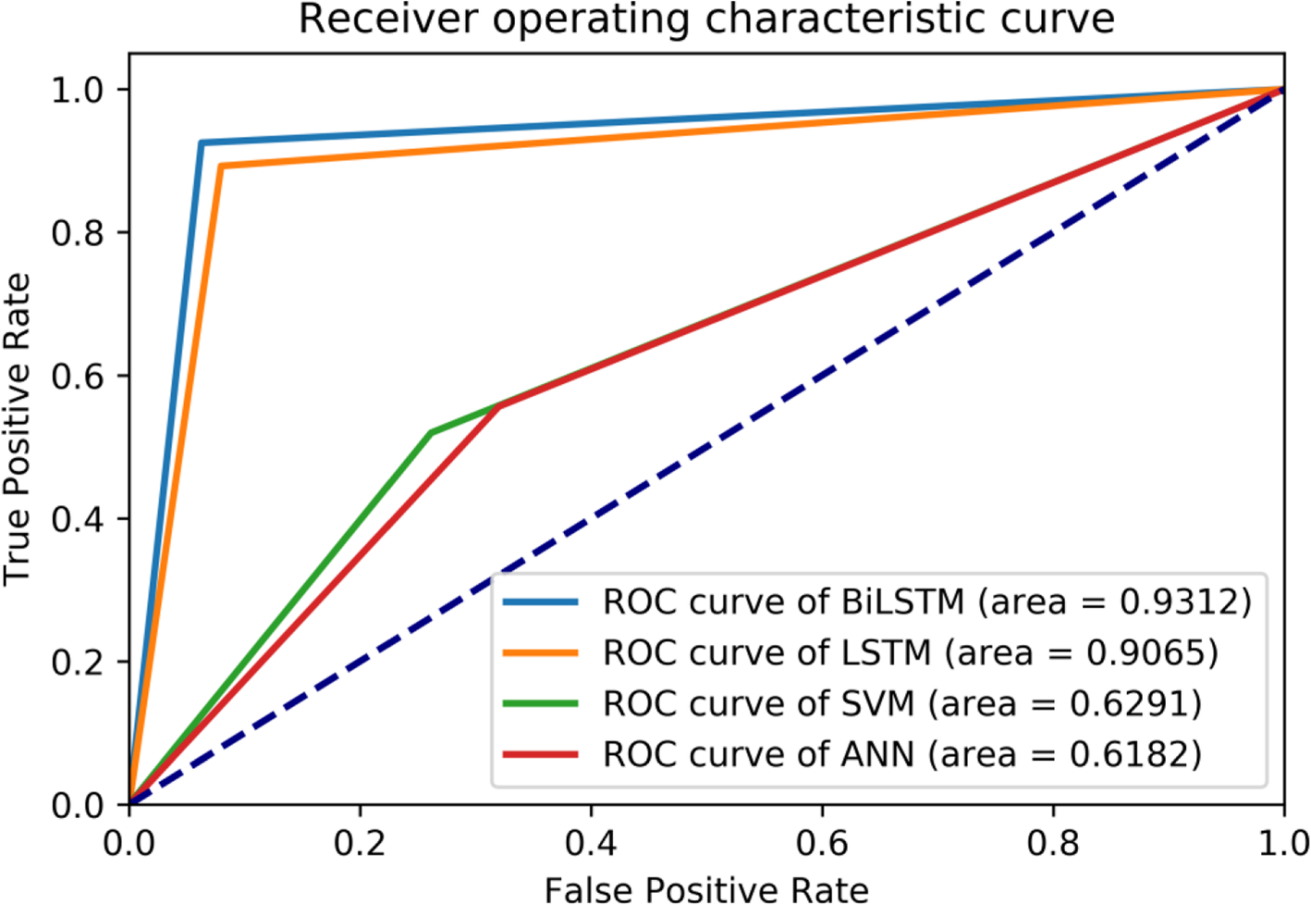
Overall recursive operating curve for forecasting models. The forecasting models include ANN, SVM, LSTM, and BiLSTM

As shown in Table 4, one can see that the deep learning-based models outperform the traditional machine learning-based models significantly. Specifically, the deep learning-based models achieve over the accuracy of 90% while the traditional machine learning-based models obtain the accuracy not over than 57%. The BiLSTM with the capability of memorizing historic information achieves the best forecasting performance with the recall of 92.51*%*, precision of 91.48*%*, accuracy of 93.21*%*, and F score of 91.98*%*. The LSTM also has the ability to memorize the historic information in sequence obtaining the accuracy of 90.85*%*, which is about 3% lower than that of the BiLSTM. The cause may lie in that the BiLSTM could capture the global information of sequences by concatenating two opposite directional information during the training stage. At the same time, we draw a figure of receiver operating characteristic curve for the best fitted forecasting model selection. As shown in Fig. 9, the traditional machine learning-based methods of the ANN and SVM perform almost the same, the AUC (area under the curve) values are around 0.6. While, the deep learning-based methods could achieve over the AUC value of 0.9. It obviously demonstrates that the deep learning-based forecasting models for one-day-forward wellness conditions outperform the traditional machine learning-based models via using single lead short ECG signals.

**Table 4:** Classification Performance of Forecasting Models with Best *Fscore*

Models		*REC*	*PRE*	*FPR*	*ACC*	*Fscore*
Deep learning-based	LSTM	89.26%	89.26%	7.96%	90.85%	89.25%
	BiLSTM	92.51%	91.48%	6.26%	93.21%	91.98%
Machine learning-based	ANN	57.74%	52.58%	37.40%	60.57%	55.03%
	SVM	51.95%	61.43%	26.14%	64.12%	56.29%

## Discussion

The best fitted forecasting model BiLSTM is selected to discuss from learned features and performance enhancement via filtering with the EWMA in this section.

### Feature visualization

**Fig. 10:**
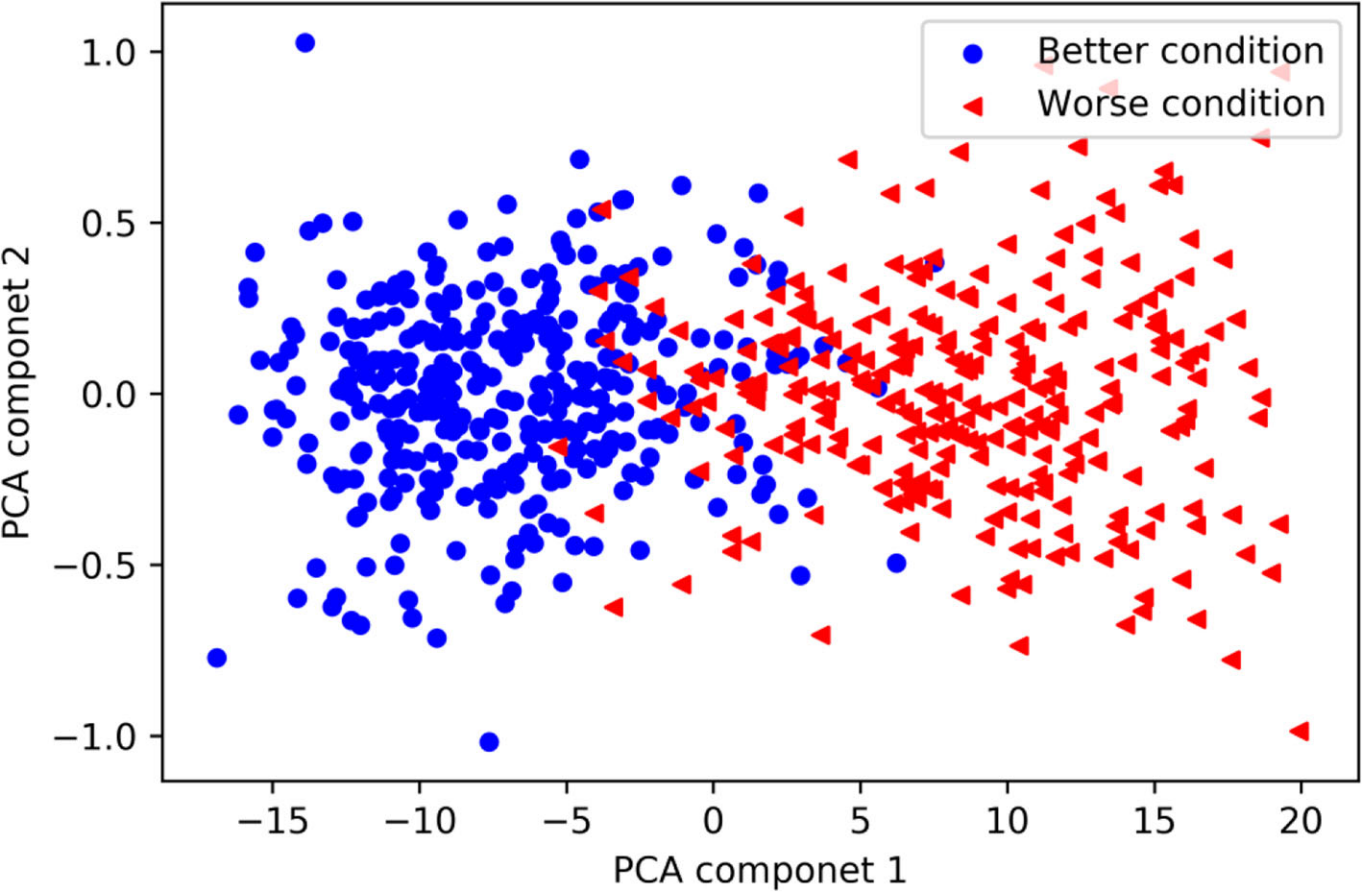
Visualization of learned features. Blue points mean better condition, red points mean worse condition

The learned features are extracted from the concatenated hidden layer of the BiLSTM based on the independent test data set. The size of the concatenated layer is 512, which is composed by two final hidden units in the opposite directional network. In order to present the learned information in a scatter plot, the dimension of learned features is reduced from 512 to 2 via the principle component analysis (PCA) method. The top two extracted principle components occupy over 98% of explained variance ratio. As shown in Fig. 10, blue points represent the better condition while the red points represent the worse points. One can see that two classes of wellness conditions can be separated linearly. It means that the BiLSTM with the ability to capture global information of an ECG signal can well solve the problem of forecasting wellness condition for community-dwelling elderly.

### Classification performance enhancement with the EWMA

**Fig. 11:**
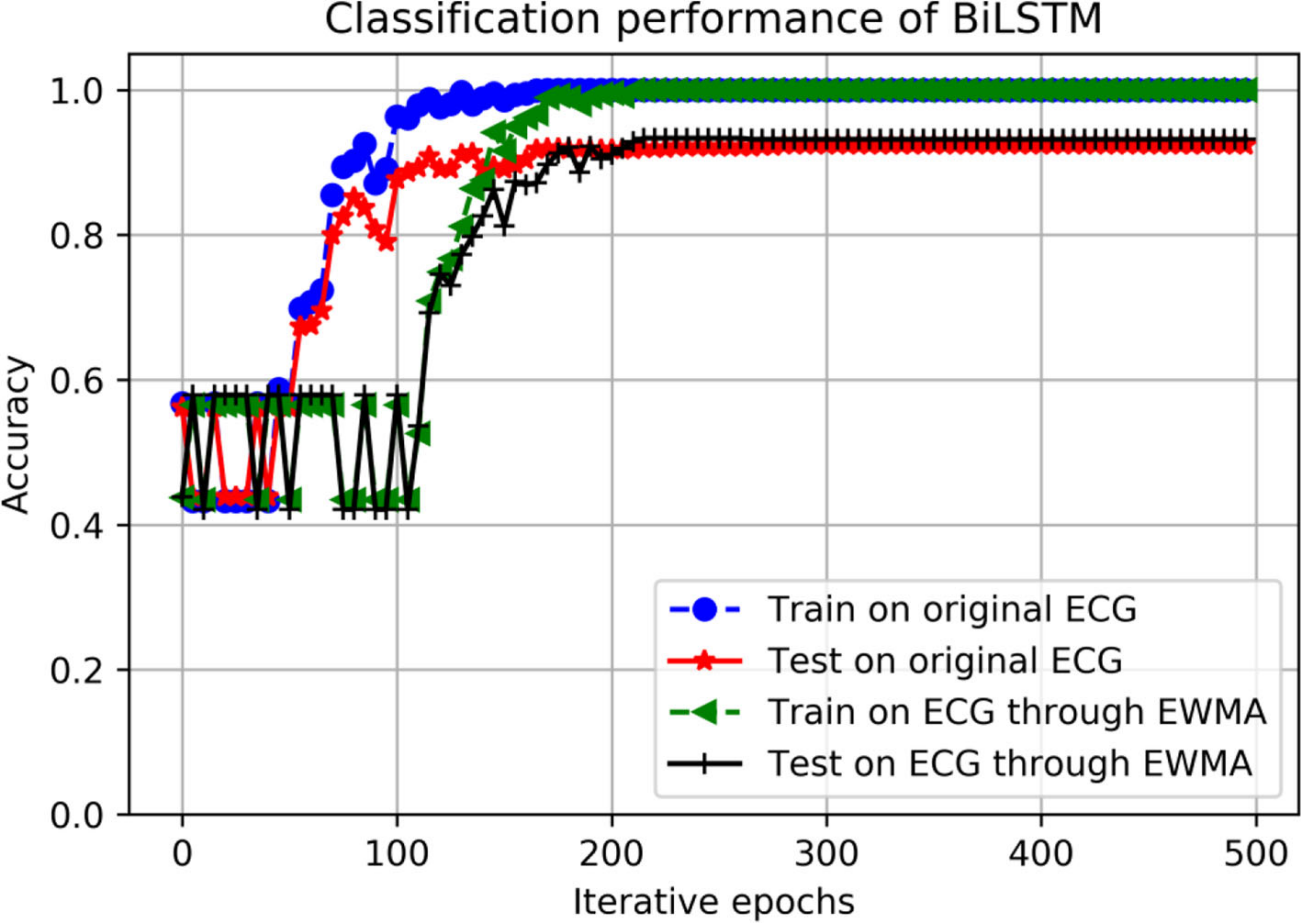
Forecasting performance of the BiLSTM. Forecasting performance of the BiLSTM between ECG preprocessing with EWMA and not

It is well known for us that noises can greatly reduce the performance of forecasting models. It is necessary to utilize a filter to remove noises from ECG signals, where may be contaminated by artifact, baseline wandering, and so on. After spectrum analysis, the noises in the ECG signals we used are mainly on high frequency, which can be shown in Fig. 2. Therefore, a filter EWMA widely applied in temporal sequential data is utilized to cancel high-frequency noises in ECG signals with the window size of 40. As shown in Fig. 11, the forecasting accuracy is vibrated around 50% until around 50 iterated epochs of the BiLSTM for original ECG signals and around 100 iterated epochs of the BiLSTM for ECG signals through the EWMA filtering. As shown in the training stage of the BiLSTM, we know that all of the training data are categorized into either class of better condition or class of worse condition. It means there is just a little difference between these two categories, which result in dramatic vibration of prediction performance in initial iterated epochs due to small parameter changes of the BiLSTM. It also demonstrates the powerful learning capability to forecast the wellness conditions for elderly. The prediction performance of the BiLSTM could meet the requirements in elderly care to avoid malignant events.

## Conclusion

In this study, we develop an approach to one-day-forward wellness forecasting for community-dwelling elderly via ECG signals analysis and modeling. The EWMA approach is employed to eliminate the influence of high-frequency noise from original ECG signals. Meanwhile, the Fisher-Yates normalization method is used to mitigate the skewness of self-evaluated wellness scores. To obtain the best fitted forecasting model for community-dwelling elderly, deep learning-based methods (LSTM and BiLSTM) and traditional machine learning-based methods (ANN and SVM) are utilized to predict the one-day-forward wellness conditions of elderly. The experiment results show that the deep learning-based methods outperform the state-of-the-art traditional machine learning-based methods. The BiLSTM achieves the best fitted forecasting performance, whose recall, precision, false prediction rate, accuracy and F score are 92.51%, 91.48%, 6.26%, 93.21%, and 91.98%, respectively. Meanwhile, visualization for the concatenated layer of the BiLSTM shows that the one-day-forward wellness conditions can be separated linearly. The best fitted BiLSTM with limited parameters could be deployed and validated on a healthcare platform. This study provides insights in terms of implementing a cost-effective approach to informing healthcare providers about health conditions of elderly in advance and taking timely interventions to reduce the risk of malignant events.

## Data Availability

Not applicable.

## Abbreviations

ANN: Artificial neural network
AUC: Area under curve
BiLSTM: Bidirectional long short-term memory network
ECG: Electrocardiogram
EHR: Electronic health records
EWMA: Exponentially weighted moving-average method
HI: Health index
IOT: Internet of things
LSTM: Long short-term memory network
RBF: Radial basis function
RNN: Recurrent neural network
ROC: Receiver operating characteristic curve
SVM: Support vector machine
